# Reproducible gene targeting in recalcitrant *Escherichia coli *isolates

**DOI:** 10.1186/1756-0500-4-213

**Published:** 2011-06-22

**Authors:** Veerle Derous, Francine Deboeck, Jean-Pierre Hernalsteens, Henri De Greve

**Affiliations:** 1 Viral Genetics Laboratory, Faculty of Science and Bio-engineering Sciences, Vrije Universiteit Brussel, Pleinlaan 2, B-1050 Brussel, Belgium; 2 Structural Biology Brussels, Faculty of Science and Bio-engineering Sciences, Vrije Universiteit Brussel, Pleinlaan 2, B-1050 Brussel, Belgium; 3 Department of Molecular and Cellular Interactions, VIB, Faculty of Science and Bio-engineering Sciences, Vrije Universiteit Brussel, Pleinlaan 2, B-1050 Brussel, Belgium

## Abstract

**Background:**

A number of allele replacement methods can be used to mutate bacterial genes. For instance, the Red recombinase system of phage Lambda has been used very efficiently to inactivate chromosomal genes in *E. coli *K-12, through recombination between regions of homology. However, this method does not work reproducibly in some clinical *E. coli *isolates.

**Findings:**

The procedure was modified by using longer homologous regions (85 bp and 500-600 bp), to inactivate genes in the uropathogenic *E. coli *strain UTI89. An *lrhA *regulator mutant, and deletions of the *lac *operon as well as the complete *type 1 *fimbrial gene cluster, were obtained reproducibly. The modified method is also functional in other recalcitrant *E. coli*, like the avian pathogenic *E. coli *strain APEC1. The *lrhA *regulator and *lac *operon deletion mutants of APEC1 were successfully constructed in the same way as the UTI89 mutants. In other avian pathogenic *E. coli *strains (APEC3E, APEC11A and APEC16A) it was very difficult or impossible to construct these mutants, with the original Red recombinase-based method, with a Red recombinase-based method using longer (85 bp) homologous regions or with our modified protocol, using 500 - 600 bp homologous regions.

**Conclusions:**

The method using 500-600 bp homologous regions can be used reliably in some clinical isolates, to delete single genes or entire operons by homologous recombination. However, it does not invariably show a greater efficiency in obtaining mutants, when compared to the original Red-mediated gene targeting method or to the gene targeting method with 85 bp homologous regions. Therefore the length of the homology regions is not the only limiting factor for the construction of mutants in these recalcitrant strains.

## Background

The entire genomic sequences of the uropathogenic *Escherichia coli *strains CFT073, UTI89 and 536 were recently determined [1-3]. At present, functions should be assigned to the relevant open reading frames (ORFs), e.g. by characterizing the appropriate mutant strains.

**Figure 1:**
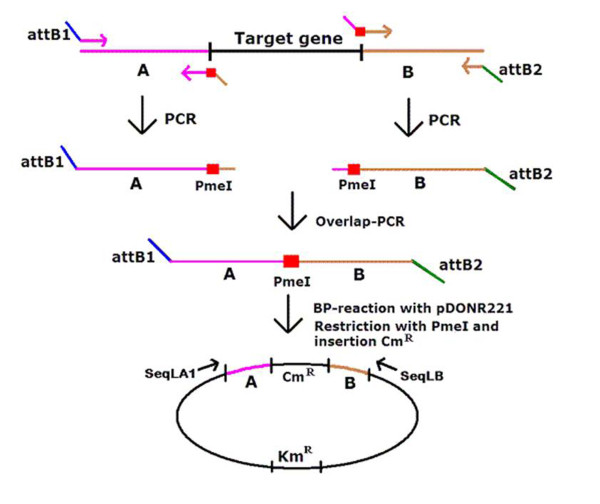
**Schematic representation of the construction of specific deletion mutants in *E. coli *UTI89**. In the first step, homology regions A and B, flanking the deletion, were amplified by PCR. Primers were designed in such a way that fragments were generated with an *attB*-site on one side and with a restriction enzyme site and an overlap region of 25-30 bp on the other side. Overlap-PCR was subsequently carried out with the *attB*-containing primers, to join regions A and B. The resulting PCR fragment was used in a BP-reaction with the pDONR221 plasmid. This gave rise to a plasmid containing the overlap-PCR fragment. After PmeI restriction of the plasmid, the chloramphenicol resistance marker *cat *was inserted. A final PCR-fragment was obtained using primers SeqLA1 and SeqLB, which are located on pDONR221. This fragment was electroporated in arabinose-induced *E. coli *strains harbouring the helper plasmid pKD46 expressing the Red recombinase.

Precise gene inactivation is an important tool in bacterial genetics. A number of allele replacement methods were developed to mutate bacterial genes. An elegant method, based on homologous recombination, mediated by the Red system of phage Lambda, is used routinely for the construction of deletion mutants in *E. coli *K-12 [4]. Pathogenic *E. coli *isolates are often more challenging to manipulate genetically than this laboratory strain. Here we report on the application and validation of a modified Red-mediated gene targeting method, in which 500-600 bp long homology regions were used to delete reproducibly specific genes in *E. coli *UTI89 (see Figure 1). This modified method allows the directed deletion of any non-essential region - one gene or an entire operon - in the chromosome, by substitution of an antibiotic resistance marker, using Red-mediated homologous recombination. Subsequently, these mutants can easily be converted into non-polar deletions. The described modified method is not only successful in *E. coli *UTI89, but is also effective in other clinical *E. coli *isolates, like avian pathogenic *E. coli *(APEC) strains. Its efficiency was compared with the original Red-mediated gene targeting procedure [4] and with another modified Red-mediated gene targeting procedure, in which longer (85 bp) homology regions were used.

## Methods

### Bacterial strains, plasmids and media

The *E. coli *strain UTI89 (serotype O18:K1:H7) [2] [GenBank:CP000243] is a human cystitis isolate. The *E. coli *strain APEC1 (serogroup O45) [5] was isolated from the peritoneum of a broiler breeder chicken. The *E. coli *strain APEC3E (serogroup O78) was isolated from the caecum of a layer chicken and the strains APEC11A and APEC16A (both serogroup O78) were obtained from the heart of layer chickens [6].

The plasmids pKD3 [GenBank: AY048742] and pKD46 [GenBank: AY048746] were described by Datsenko and Wanner [4]. Plasmid pDONR221 was purchased from Invitrogen. The plasmid pEHEC356 [7] was used as a control in the transformation experiments.

Bacteria were grown routinely in LB broth and on LB agar plates [8]. The medium was supplemented with chloramphenicol (25 μg/ml), carbenicilline (100 μg/ml) or kanamycin (25 μg/ml) if needed.

For electroporation, salt-optimized carbon broth (SOC) was used [9].

Phosphate Buffered Saline (PBS) was as described [10].

### Modified Red-mediated gene targeting method, using 500-600 bp homology regions (see Figure 1)

#### Amplification and purification of DNA-fragments

Polymerase chain reactions (PCR) were carried out in an Applied Biosystems 2720 Thermal Cycler using Ex Takara DNA Polymerase. Annealing and elongation temperatures were 55°C and 68°C respectively. Elongation time was 1 minute per 1000 bp. The primers used in the PCR reactions are listed in Table 1. PCR-fragments were purified using the Qiaquick PCR Purification Kit (Qiagen). PCR products and purified PCR fragments were analyzed by electrophoresis on 1.0% agarose gels.

**Table 1:** List of primers

**Primer **	**Sequence (5' - 3')**
SeqLA1	CTCTCGCGTTAACGCTAGCATGGAT

SeqLB	GTAACATCAGAGATTTTGAGACAC

P1	GTGTAGGCTGGAGCTGCTTC

P2	CATATGAATATCCTCCTTAG

AttB1LrhA	GGGGACAAGTTTGTACAAAAAAGCAGGCTTACGGCAGATGGACGCCACATCGATT

LrhAPmeI-1	AGTATGAGCCGCCAGTAAGTGATAATATATGGTTTAAACTCGACGGACGATAGATAATT

LrhAPmeI-2	ATTGTCTCAGGAATTATCTATCGTCCGTCGAGTTTAAACCATATATTATCACTTACTGGCGGCTCA

AttB2LrhA	GGGGACCACTTTGTACAAGAAAGCTGGGTCTGCTCTTGATGCCGCCTCACCATT

LrhA5	CAGTGATGAGCGATGACTTCAGTG

LrhA6	TAGAAGATTACTTTGCCTAACATA

AttB1Lac	GGGGACAAGTTTGTACAAAAAAGCAGGCTTACTACGGCAATGCACTCCTATAA

LacPmeI-1	TAACAATTTCACAGGATACAGCTATGGTTTAAACATAAGCAAAATTGCCTGATGCGCTCCGCTT

LacPmeI-2	AAGCGGAGCGCATCAGGCAATTTTGCTTATGTTTAAACCATAGCTGTATCCTGTGTGAAATTGTTA

AttB2Lac	GGGGACCACTTTGTACAAGAAAGCTGGGTATAAATATCTC ACACGCAATCAAATTCA

LacZYA-1	TTGTTGGGGCGATTCCGCATTTTGAATTTA

LacZYA-2	GTCAGTGGGCTGATCATTAACTAT

AttB1Type1	GGGGACAAGTTTGTACAAAAAAGCAGGCTAT CTCCAGGAAATACACAGTCTGAAA

Type1PmeI-1	TACCTGCATTAGCAATGCCCTGTGA TTTCTGTTTAAACCATCGTTTTCCCTTATAATTACAGACGCGCACTA

Type1PmeI-2	TAGTG CGCGTCTGTAATTATAAGGGAAAACGATGGTTTAAACAGAAATCACAGGGCATTGCTAATGCAGGTA

AttB2Type1	GGGGACCACTTTGTACAAGAAAGCTGGGTGTACCAGCG CCAGGTCTGTTCCATGATTT

Type1-1	GAAATATGTTTCCTGGTTTTTGGCTTGTAA

Type1-2	ACGACAGACCACACCAGGCCTGCGTCTT

Type1DW1	TATTGCTAACCCAGCACAGCTAGTGCGCGTCTGTAATTATAAGGGAAAACG *TTGAGCGATTGTGTAGGCTGGAGCTGCTTC*

Type1DW2	GTTTTAGCTTCAGGTAATATTGCGTACCTGCATTAGCAATGCCCTGTGATTTCT *TTAGCCATGGTCCATATGAATATCCTCCTTAG*

LrhADW1	GTGTGCACAGCATTAACCAGCTCAGTATGAGCCGCCAGTAAGTGATAATA *TGTGTAGGCTGGAGCTGCTTC*

LrhADW2	CAGCGGCTCGTTTTTTACACTATTGTCTCAGGAATTATCTATCGTCCGTC *CATATGAATATCCTCCTTAG*

LacZDW1	GTATGTTGTGTGAAATTGTGAGCGAATAACAATTTCACACAGGATACAGCT *TTGAGCGATTGTGTAGGCTGGAGCTGCTTC*

LacZDW2	TGAAATTGTAGGCCTGATAAGCGGAGCGCATCAGGCAATTTTGCTTATTTA *TTAGCCATGGTCCATATGAATATCCTCCTTAG*

Type1DWL1	CGTAAGCTGACGAATCAGCAGGAATAATCGCTAGGGACCTAAGAATTAGCATGATAATAGCCACTAAGAAATTACTGCGCTCCA*TGTGTAGGCTGGAGCTGCTTC*

Type1DWL2	TTATCTGGCCTACAAAGGGCTAACGTGCAGGTTTTTAGCTTCAGGTAATATTGCGTACCAGCATTAGCAATGTCCTGTGATTTCT*CATATGAATATCCTCCTTAG*

LrhADWL1	GGGAGGCACATTACAAATGGAATTGCTTGTTTGTGTGTGCACAGCATTAACCAGCTCAGTATGACCCGCCAGTAAGTGATAATA*TGTGTAGGCTGGAGCTGCTTC*

LrhADWL2	CTAAAAAAAAGCCGCTGGGGTTTAAAACACCCCCAGCGGCTCGTTTTTTACACTATTGTCTCAGGAATTATCTATCGTCCGTCGAC*CATATGAATATCCTCCTTAG*

LacZDWL1	TTAGGCACCCCAGTCTTTAC ACTCTATGTG TCCGGCTCGT ATGTTGTGTG AAATTGTGAGCGAATAACAATTTCACACAG GATACAGCT*TTGAGCGATT GTGTAGGCTG GAGCTGCTTC *

LacZDWL2	GCGGCGTGAACACCTTATCC GGCCTACGTA GATCTCTGAA ATTGTAGGCC TGATAAGCGG AGCGCATCAGGCAATTTTGC TTATTTA*TTAGCCATGGTCCATA TGAATATCCT CCTTAG *

The AttB1 and AttB2 sites are underlined. P1 and P2 sites are italicized.

#### Construction of intermediate gene replacement vectors

First, two 500-600 bp homology regions ('A' and 'B'), surrounding the targeted sequence, were amplified from total genomic DNA of *E. coli *UTI89. Primers (see Table 1) are designed in such a way that the PCR-fragments A and B have an overlap of approximately 25-30 bp. After purification of the PCR-fragments A and B, they were joined by overlap-PCR, which was carried out with the two 'outside' primers containing the *attB*-sites of the Gateway^® ^cloning system. The Gateway^® ^Technology (Invitrogen) is a universal cloning method providing a rapid and efficient way to move DNA sequences into multiple vector systems. The technology is based on the site-specific recombination system of bacteriophage lambda. This recombination occurs between site-specific *att*achment (*att*) sites and is catalyzed by a mixture of enzymes. The components of the lambda recombination system were modified to improve the specificity and efficiency of the system.

The resulting PCR-fragment comprises homology regions A and B, flanked by *attB*-sites, and has a unique PmeI restriction enzyme site situated between the two homology regions. The *attB *sites are used for a site-specific BP-recombination reaction with the *attP *sites of the pDONR221 vector, using the Gateway^® ^technology (Invitrogen). After transformation to CaCl_2_-competent *E. coli *DH5α cells [11], clones were selected on LB medium containing kanamycin. Kanamycin-resistant clones were screened by PCR using primers SeqLA1 and SeqLB, which are flanking the insert on pDONR221.

Plasmid DNA was prepared from sequenced clones, using the GeneElute™ Plasmid Miniprep Kit (Sigma) and cut with restriction enzyme PmeI. Subsequently, the *cat *gene of plasmid pKD3, flanked by FRT sites, was amplified with primers P1 and P2 [4] and made blunt using T4 DNA polymerase. Ligation of the 1034 bp *cat *fragment into the PmeI-digested plasmid DNA was performed with the Rapid DNA Ligation Kit (Roche). After transformation into *E. coli *DH5α competent cells, transformants were selected on chloramphenicol-containing LB medium.

#### Gene replacement using PCR fragments

PCR-fragments carrying both homology regions A and B, flanking the P1-FRT-*cat*-FRT-P2 sequence, were amplified with primers SeqLA1 and SeqLB, using the pDONR221 clones as template. After purification, about 1 μg of this DNA was introduced by electroporation (Bio-Rad Gene Pulser apparatus, settings: 25 μF and 2.5 kV; Pulse controller set to 200 Ω) into arabinose-induced *E. coli *harbouring the Red recombinase system of Lambda, encoded by the helper plasmid pKD46 [4]. After electroporation, cells were immediately resuspended in SOC medium and incubated at 37°C for at least one hour. After incubation, aliquots were spread on LB agar plates with chloramphenicol and incubated at 37°C. Chloramphenicol resistant colonies, that were kanamycin sensitive, were checked by PCR, using primers located outside the flanking homology regions A and B (see Table 1, primers pairs LrhA5/LrhA6, LacZYA-1/LacZYA-2 or Type1-1/Type1-2), and primers flanking the *cat *gene (primers P1 and P2). The transformants, that became simultaneously chloramphenicol and kanamycin resistant, were shown to carry the whole pDONR221-derived template plasmid, present in trace amounts in the PCR fragments used for electroporation, instead of an insertion of the *cat *gene.

#### Elimination of the antibiotics resistance marker

The mutants carry the same P1-FRT-*cat*-FRT-P2 insert as in the original Red recombinase-based method [4]. Therefore the *cat *gene can be easily removed by expressing the FLP recombinase. This FLP, originating from yeast, recognizes the FRT-sites and mediates a site-specific recombination, so that the antibiotic resistance gene is eliminated leaving the same P1-FRT-P2 scar that has no polar effect on gene expression [4]. The *cat *gene was eliminated by use of the temperature sensitive helper plasmid pCP20 [4], encoding the FLP recombinase.

For this aim, the plasmid PCP20 was introduced by electroporation in the strain of which the antibiotic resistance marker had to be eliminated. After expression at 28°C for 2 hours, the culture was plated on LB medium with carbenicilline (100 μg/ml) and incubated overnight at 28°C. After purification of the colonies on the same medium, these were purified a second time on LB medium without antibiotics and incubated at 42°C. The resulting colonies, growing on LB medium but not on LB with carbenicilline and on LB medium with chloramphenicol, lost the helper plasmid pCP20 and the *cat *gene.

### Original Red-mediated gene targeting method and modified method, using 85 bp homology regions

PCR-fragments, carrying homology regions flanking the *type 1*, *lrhA *or *lac *genes and the *cat *gene of plasmid pKD3 were purified and about 1 μg of this DNA was introduced by electroporation into arabinose-induced *E. coli *strains, harbouring pKD46. Primer pairs LrhADW1/2, LacZDW1/2 and Type1DW1/2 (Table 1) were used to amplify the regions flanking *lrhA*, *lac *and *type 1 *genes respectively, for the original Red-mediated gene targeting method [4]. Primer pairs LrhADWL1/2, LacZDWL1/2 and Type1DWL1/2 (Table 1) were used to amplify the regions flanking *lrhA*, *lac *and *type 1 *genes respectively, for the Red-mediated gene targeting method with 85 bp homology regions.

Electroporation for the side-by-side comparison was carried out into the same batch of arabinose-induced cells. To test the transformation efficiency of the strains, the plasmid pEHEC356 was transformed in the same batch of arabinose-induced *E. coli *harbouring the Red recombinase expressing plasmid pKD46.

### Phenotypic assays

#### Motility test of *E. coli *strains

Motility of the *E. coli *strains was investigated by transferring mutant and wild type colonies onto the same plate containing LB with 0.3% Select agar (Sigma Aldrich) with a toothpick. After incubation at 30°C for 12-16 hours, the diameter of the colonies was observed [12].

#### Agglutination of yeast cells

Adhesion mediated by type 1 fimbriae was tested by agglutination of commercial baker's yeast cells (*Saccharomyces cerevisiae*) on glass slides [13]. Bacteria were grown statically in LB broth at 37°C for 72 hours. Aliquots of washed bacterial suspensions were incubated with a 2% yeast suspension in PBS and put on a glass microscope slide. Agglutination was investigated using a stereomicroscope. As a control, 1% D-mannose (final concentration) was used to inhibit the agglutination mediated by type 1 fimbriae.

#### Lactose fermentation assay

Lactose fermentation was tested on MacConkey medium supplemented with 1% lactose. Lac mutants grow as white colonies, while wild type Lac^+ ^colonies are red.

### Statement of ethical approval

The present study was approved by the Ethical Committee for Animal Experiments of the Vrije Universiteit Brussel (project number 06-219-3) and performed following all national legislation and institutional policies.

## Results and discussion

### Gene replacement in *E. coli *UTI89

#### Deletion of the *lrhA *regulator gene

The LysR-type transcriptional regulator LrhA (LysR homologue A) controls genes involved in flagellation, motility and chemotaxis [14]. An *lrhA *mutant of *E. coli *K-12 showed an increased number of flagellae compared to the wild type. Motility of uropathogenic *E. coli *(UPEC) plays an important role in colonization of the urinary tract [15]. UPEC presumably utilize flagellum-mediated motility during urinary tract infections to ascend to the upper urinary tract and the kidneys [16]. The transcriptional regulator LrhA also regulates the expression of type 1 fimbriae: inactivation of *lrhA *in *E. coli *K-12 results in an increased expression of type 1 fimbriae and increased biofilm formation [13].

**Figure 2:**
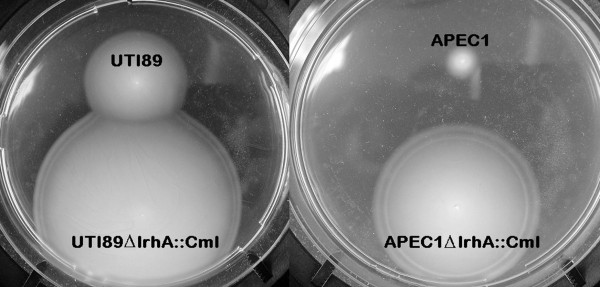
**Motility of wild type and *lrhA *mutant *E. coli *UTI89 and APEC1**. Motility of the *E. coli *strains was investigated by transferring mutant and wild type colonies onto the same plate containing LB with 0.3% agar, with a sterile toothpick. After incubation, the diameter of the colonies was compared [12]. The motility of the *lrhA *mutants is much higher than the motility of the corresponding wild type strains.

Deletion of a single functional gene in *E. coli *UTI89, by use of the modified Red-mediated gene targeting procedure, in which 500-600 bp long homology regions were used to delete genes, was tested by construction of an UTI89Δ*lrhA*::Cm^R ^mutant. The motility of the UTI89Δ*lrhA*::Cm^R ^mutant, in semi-solid LB medium, was much higher than the motility of wild type UTI89 (see Figure 2). This was expected, because Blumer *et al. *[13] reported increased motility of an *lrhA *mutant of *E. coli *MG1655 and UPEC strain 536, due to the increased number of flagellae per cell.

Type 1 fimbriae of *E. coli *mediate the adherence to mannose-containing receptors. Adherence mediated by fimbriae is important for the virulence of uropathogenic *E. coli *(UPEC), and the expression of type 1 fimbriae is required for colonization of the urinary tract [17,18]. Adhesion, mediated by type 1 fimbriae, was monitored by the agglutination of yeast cells. The agglutination caused by the UTI89Δ*lrhA*::Cm^R ^mutant was significantly stronger, compared to the wild type (data not shown). Addition of mannose abolished agglutination, demonstrating that agglutination is mannose-specific and thus depends on the expression of type 1 fimbriae. The UTI89Δ*lrhA*::Cm^R ^mutant also produced more biofilm than wild type UTI89 (data not shown). These results confirm the phenotype of the UTI89Δ*lrhA*::Cm^R ^mutant and show that the described method allows the site-specific deletion of single genes in the *E. coli *UTI89 genome.

A side-by-side comparison between the original Red-mediated gene inactivation method [4], a modified version of the original method with longer (85 bp) homology regions and the described overlap PCR-based method using 500-600 bp homology regions was carried out (See Table 2). UTI89Δ*lrhA*::Cm^R ^mutants were obtained with the three methods.

**Table 2:** Side-by-side comparison between the original and modified Red-mediated gene targeting methods.

	UTI89	APEC1	APEC3E	APEC11A	APEC16A
**Original Red-mediated gene targeting method**					

Deletion of *type 1 *fimbriae cluster	0	0	0	1	0

Deletion of *lac *operon	2	0	0	0	0

Deletion of *lrhA *regulator gene	1	0	0	0	0

**Modified Red-mediated gene targeting method using 85 bp long homology regions**					

Deletion of *type 1 *fimbriae cluster	2	1	0	0	0

Deletion of *lac *operon	1	4	0	0	0

Deletion of *lrhA *regulator gene	1	2	2	1	0

**Modified Red-mediated gene targeting method using 500-600 bp long homology regions**					

Deletion of *type 1 *fimbriae cluster	2	0	0	0	0

Deletion of *lac *operon	18	2	0	5	0

Deletion of *lrhA *regulator gene	1	1	0	0	0

The number of chloramphenicol resistant and kanamycin sensitive colonies confirmed by PCR is indicated.

#### Deletion of the *lac *operon

The *lac *operon, consisting of three adjacent genes (*lacZ*, *lacY *and *lacA*) is required for the transport and catabolism of lactose in *E. coli *[19]. UTI89Δ*lacZYA*::Cm^R ^mutants were constructed in the same way as described for the UTI89Δ*lrhA*::Cm^R ^mutant, by use of the modified Red-mediated gene targeting procedure, in which 500-600 bp long homology regions were used. The primer pairs AttB1Lac/LacPmeI-1 and LacPmeI-2/AttB2Lac were used to amplify the homology regions flanking the *lac *operon. Finally, 18 out of 20 of the chloramphenicol resistant UTI89 tranformants were kanamycin sensitive and produced the expected PCR fragments. All of these UTI89Δ*lacZYA*::Cm^R ^grew as white colonies on MacConkey medium supplemented with lactose, while wild type UTI89 produced red colonies. The *cat*-resistance marker was successfully eliminated by introduction of the pCP20 helper plasmid, giving rise to UTI89Δ*lacZYA*.

A comparison between the different methods was carried out (See Table 2). UTI89Δ*lacZYA*::Cm^R ^mutants were obtained with the three methods. As was the case for the UTI89Δ*lrhA*::Cm^R ^mutants, the length of the different homology regions used, seemed not to be the determining factor for obtaining mutants.

#### Deletion of the *type 1 *fimbriae cluster

Adherence of UPEC to the bladder epithelial cells is mediated by type 1 fimbriae and is crucial to prevent washout of the pathogenic bacteria by the flow of urine [17] and for uptake of the bacteria into the bladder epithelial cells. The *type 1 *fimbriae of UPEC are encoded by the *fim *gene cluster, consisting of nine genes: *fimB*, *fimE*, *fimA*, *fimI*, *fimC*, *fimD*, *fimF*, *fimG *and *fimH*. For the construction of the *type 1 *fimbrial mutants, primer pairs AttB1Type1/Type1PmeI-1 and Type1PmeI-2/AttB2Type1 were used to amplify the homology regions flanking the *type1 *gene cluster. Two out of 6 chloramphenicol resistant UTI89 transformants were kanamycin sensitive and were confirmed by PCR. These UTI89Δ*fimBEAICDFGH*::Cm^R ^mutants did not agglutinate yeast cells. The *cat*-resistance marker was successfully eliminated by introduction of the pCP20 helper plasmid, giving rise to UTI89Δ*fimBEAICDFGH*.

UTI89Δ*fimBEAICDFGH*::Cm^R ^mutants were obtained with both modified versions of the Red-mediated gene targeting method, but no mutant was obtained with the original Red-mediated gene inactivation method (see Table 2).

### Gene replacement in avian pathogenic *E. coli*

We investigated whether the overlap PCR-based method was also functional in the avian pathogenic *E. coli *strain APEC1 (see Table 2). The APEC1Δ*lrhA*::Cm^R ^and APEC1Δ*lacZYA*::Cm^R ^mutants were successfully constructed and confirmed by PCR. A motility test with the APEC1Δ*lrhA*::Cm^R ^mutant confirmed that its motility is much higher than the motility of the wild type APEC1 control (see Figure 2). On MacConkey medium supplemented with lactose, APEC1Δ*lacZYA*::Cm^R ^mutants grew as white colonies, while wild type APEC1 produced red colonies (data not shown). This confirms that the modified Red-mediated gene targeting method, using long (500-600 bp) homology regions can also be applied in this APEC strain. The *cat*-resistance marker was also successfully eliminated by introduction of the pCP20 helper plasmid, giving rise to APEC1Δ*lacZYA*.

Mutants were obtained with both modified versions of the Red-mediated gene targeting method, but no mutants were obtained with the original version of the Red-mediated gene inactivation method Although APEC1 showed a 5-times higher number of transformants with the plasmid pEHEC356 than *E. coli *UTI89, this did not lead to a 5-times larger number of mutants than in *E. coli *UTI89. Therefore, the efficiency of transformation of these strains is not the determining factor (see Table 2).

We also investigated whether the overlap PCR-based method was functional in the avian pathogenic *E. coli *strains APEC3E, APEC11A and APEC16A (See Table 2). For APEC3E we only obtained APEC3EΔ*lrhA*::Cm^R ^by use of the Red-mediated procedure with 85 bp homology regions. No other mutants were found in APEC3E by any of the other methods. APEC11AΔ*lrhA*::Cm^R ^mutants were also only obtained with the Red-mediated procedure with 85 bp homology regions, an APEC11AΔ*type1*::Cm^R ^mutant was only obtained with the original Red-mediated procedure with 50 bp homology regions and APEC11AΔ*lacZYA*::Cm^R ^mutants could only be obtained with the overlap PCR-based procedure with 500-600 bp long homology regions. For APEC16A, it was not possible to construct mutants by any of the three methods used. Nevertheless, transformation of this strain with plasmid DNA was possible at a low frequency (150 colonies were obtained using 1 ng of plasmid pEHEC356 DNA for electroporation). An attempt to inactivate possible restriction-enzyme activity by heating at 50°C for 30 min [20] did not lead to any results in the gene targeting and increased the number of pEHEC356 transformants to 200 colonies per ng of plasmid pEHEC356 DNA.

## Conclusions

Methods for disrupting *E. coli *chromosomal genes, based on an efficient method for *E. coli *K-12 [4] were tested in clinical UPEC and APEC isolates. Similar Red-based technology has been successfully used for other Gram-negative bacteria, like for example *Salmonella *[21], *Pseudomonas *[22], *Yersinia *[23], *Shigella *[24] and *Vibrio cholerae *[25]. Alterations of the gene disruption method are occasionally required [22,25,26]. These approaches mostly rely on the expression of a phage recombinase in the bacterial strain. We initially found that deleting specific genes in *E. coli *UTI89, by the original Red-mediated gene targeting method [4], occurred at a low and variable frequency and was frequently unsuccessful. An alternative strategy for constructing specific mutants, by phage P1-mediated transduction of insertions from *E. coli *K-12 to *E. coli *UTI89, was also found to be inadequate. The transduction frequency was invariably low and some experiments were unsuccessful. In addition, transduction is impossible when genes are targeted which are not present in the *E. coli *K-12 genome. We also noticed that a mutant constructed by transduction of an insertion from the Keio collection in *E. coli *K-12 [12] to UTI89 had a different phenotype than the mutant constructed in UTI89 by the present procedure (data not shown). This is presumably due to the transduction of additional polymorphisms from *E. coli *K-12 to UTI89.

We modified the Red-mediated procedure in such a way that larger regions of homology (500 - 600 bp) were generated. Our results demonstrate that mutations of specific genes and also deletions of larger chromosomal regions, like for example the *lac *or the *type 1 *operon in *E. coli *UTI89 could be reproducibly obtained. Besides the deletions reported in the present article, mutations in 15 other genes were easily obtained at the first attempt. The method is not only successful in uropathogenic *E. coli *UTI89, but it is also effective in other *E. coli *isolates such as the APEC strain APEC1.

When a final side-by-side comparison was made with the original Red-mediated gene targeting method [4], using 50 and 85 bp long regions of homology, it was found that the length of the homology regions is not the only determining factor for obtaining mutants. In this experiment, the 500-600 bp long homology method was not significantly more efficient in *E. coli *UTI89 and different APEC strains. This again illustrates our observations on the low reproducibility of Red-mediated gene targeting in clinical isolates. Although the use of 500-600 bp long homology regions increases the reproducibility of the method, other factors, possibly the expression level of the Red proteins at the time of transformation or the efficiency of the electroporation procedure facilitate the targeting in some experiments.

## Abbreviations

*Cat*: chloramphenicol acetyltransferase; Cm: chloramphenicol; Km: kanamycin.

## Authors' contributions

VD participated in the design of the study, carried out the DNA manipulation and genetic procedures, wrote the manuscript and drew the figures. FDB performed the experiments on motility, agglutination of yeast cells and lactose fermentation. HDG and J-PH coordinated the study and helped to write the manuscript. All authors read and approved the final manuscript.
